# Gut-derived small extracellular vesicles support trained innate immune tolerance in murine microglial cells

**DOI:** 10.3389/fimmu.2026.1858954

**Published:** 2026-08-06

**Authors:** Trim Lajqi, Natascha Köstlin-Gille, Cahit Birdir, Janine Hebel, Ilir Miftari, Reinhard Bauer, Sotirios G. Zarogiannis, Charlotta Funaya, Christian Gille, Stefanie Dietz-Ziegler

**Affiliations:** 1 Department of Neonatology, Medical Faculty Heidelberg, University of Heidelberg, Heidelberg, Germany; 2 Department of Gynaecology and Obstetrics, Medical Faculty Heidelberg, University of Heidelberg, Heidelberg, Germany; 3 Department of Neonatology, University of Tübingen, Tübingen, Germany; 4 Department of Urology, University Clinical Center of Kosovo, Faculty of Medicine, University of Prishtina, Pristina, Kosovo; 5 Institute of Molecular Cell Biology, Jena University Hospital, Jena, Germany; 6 Department of Physiology, School of Health Sciences, Faculty of Medicine, University of Thessaly, Larissa, Greece; 7 Electron Microscopy Core Facility (EMCF), University of Heidelberg, Heidelberg, Germany

**Keywords:** epigenetics, extracellular vesicles, gut, inflammation, metabolism, microglia, migration, phagocytosis

## Abstract

**Introduction:**

Microglia are the resident immune cells of the central nervous system (CNS) that maintain tissue homeostasis and contribute to the pathogenesis of neuroinflammatory disorders. As innate immune cells, microglia can acquire memory-like states that exert long-term effects on CNS function and disease susceptibility. Increasing evidence highlights a dynamic interaction between the gut microbiota and the CNS, shaping microglial maturation and responsiveness throughout life. In addition to soluble microbial metabolites, gut-derived extracellular vesicles (EVs), including vesicles of microbial origin, have emerged as important mediators of microbiota–host communication capable of modulating brain homeostasis and inflammatory signaling; however, their role in programming microglial immune memory remains unclear.

**Methods:**

Here, we examined whether gut-derived small EVs influence memory-like features of primary murine microglia *in vitro*. Microglia were primed with small EVs followed by a secondary lipopolysaccharide (LPS) challenge, and inflammatory signaling, metabolic activity, epigenetic markers, and effector functions (migration and phagocytosis) were assessed.

**Results:**

Small EV priming followed by secondary LPS challenge induced a trained innate immune tolerance phenotype characterized by reduced pro-inflammatory mediator release and attenuated TLR2/4–MyD88–p38 MAPK signaling. This tolerant state was accompanied by suppressed glycolytic activity and decreased levels of activating histone H3 marks, indicating coordinated metabolic and epigenetic reprogramming. Notably, despite diminished inflammatory signaling, small EV–primed microglia displayed enhanced migratory and phagocytic capacities associated with increased ERK1/2 activation.

**Discussion:**

Together, these findings indicate that gut-derived small EVs can imprint memory-like programs in microglia that restrain inflammatory activation while preserving essential effector functions *in vitro*, suggesting a mechanism by which microbiota–brain communication may shape neuroinflammatory responses.

## 1 Introduction

Microglia represent the resident macrophage population of the CNS and serve as its primary innate immune effector cells (1–3). During early brain development, microglia contribute to circuit formation, pathogen clearance, and the maintenance of CNS homeostasis throughout life (4–6). They regulate neuronal maturation, synapse formation and pruning, and neuroinflammatory processes through directed migration, phagocytosis, antigen presentation, and cytokine release. Microglia exhibit substantial functional plasticity, allowing their activation states to adapt in response to repeated or chronic environmental cues (7–9). Increasing evidence indicates that following activation, microglia do not invariably return to a naïve resting state but may persist in a primed or post-activated phenotype, with potential long-term implications for CNS homeostasis and disease susceptibility (5, 10). While transient microglial activation is required for host defense and tissue repair, prolonged or dysregulated activation has been implicated in the progression of neuroinflammatory disorders (11–13).

Although immunological memory was historically attributed to adaptive immune cells, it is now well established that innate immune cells can acquire long-lasting functional (memory-like) reprogramming after an initial challenge (14–16). Depending on the nature, intensity, and duration of the priming stimulus, as well as the differentiation state of the cells, this process may result in either enhanced responsiveness, termed trained innate immunity, or in trained innate immune tolerance, characterized by attenuated inflammatory output upon secondary stimulation challenges (14, 15, 17). Such memory-like adaptations are maintained through coordinated changes in signaling pathways, metabolic activity, and chromatin structure that stabilize specific transcriptional programs (15, 18–21). Given their lifelong exposure to fluctuating environmental signals, microglial memory-like adaptations are increasingly recognized as important modulators of neuroinflammatory responses and disease progression (7, 22, 23). In this context, both microbial pathogen-associated molecular patterns (PAMPs) and endogenous danger signals have been shown to induce persistent microglial reprogramming relevant to neurological pathology (24–27).

The gut microbiota has emerged as an important regulator of CNS immunity through bidirectional communication along the gut–brain axis (28, 29). Microbiota-derived factors, including short-chain fatty acids (SCFAs) such as acetate, can cross epithelial and vascular barriers and influence microglial maturation, metabolism, and immune responsiveness (30, 31). Beyond soluble metabolites, gut-derived EVs, including vesicles of microbial origin, particularly small EVs (30–150 nm) that carry bioactive proteins, lipids, and nucleic acids, have emerged as important mediators of microbiota–host communication capable of influencing microglial development, homeostatic regulation, and inflammatory responsiveness within the CNS (32–35). However, the contribution of gut-derived small EVs to memory-like immune programming in microglia, particularly trained innate immune tolerance, remains poorly defined.

In the present study, we investigated whether small EVs derived from the gut can induce trained innate immune tolerance in primary murine microglia and characterized the associated inflammatory, metabolic, functional, and epigenetic changes. Our findings show that microglial priming with small EVs, followed by a secondary lipopolysaccharide (LPS) challenge, resulted in a tolerant anti-inflammatory phenotype, with reduced production of pro-inflammatory mediators (e.g., TNF-α, IL-6, IL-1β, ROS) associated with decreased activation of the Toll-like receptor (TLR)–mitogen-activated protein kinase (MAPK) pathway. These effects were accompanied by decreased glycolytic activity with declined L-lactate levels, and decreased expression of histone H3 marks associated to active chromatin (i.e., H3K4me3, H3K27ac, H3K18la). Despite this restrained inflammatory profile, small EV–trained microglia exhibited enhanced migratory and phagocytic capacities *in vitro*, mediated particularly by extracellular signal–regulated kinase (ERK) 1/2 pathway.

## 2 Materials and methods

### 2.1 Animals

Primary microglial cells were derived from neonatal C57BL/6J mice at postnatal days 0–3 (P0–P3) obtained from a locally maintained inbred colony. Animals were housed under standardized conditions with a 12 h light/dark cycle and unrestricted access to food and water. All animal procedures were conducted in strict accordance with Directive 2010/63/EU of the European Parliament and Council for the protection of animals used for scientific purposes.

Tissue and organ collection protocols were reviewed and approved by the Regional Council of Karlsruhe (Regierungspräsidium Karlsruhe, Germany) under authorization number Az T-02/20, and Regional Council of Tübingen (Regierungspräsidium Tübingen, Germany) under authorization number K 03/23 G.

### 2.2 Primary microglia isolation procedure

Primary microglial cells were isolated from the cerebral cortices of neonatal C57BL/6J mice, pooling 6–10 brains from both sexes, following previously established protocols (36, 37). Briefly, decapitated neonates were placed in Petri dishes containing ice-cold phosphate-buffered saline (PBS). The meninges and brainstem were carefully removed using fine scissors, and the cortices and hippocampi were dissected and collected in 15-ml tubes containing sterile PBS. Tissue fragments were enzymatically dissociated in Dulbecco’s Modified Eagle’s Medium – DMEM (Cat. D6429, Sigma-Aldrich), supplemented with 2.5% trypsin (Gibco, Cat. 15090046) and 2 mg/ml DNase I (AppliChem, Cat. A3778) for 30 min at 37 °C in a 5% CO_2_ incubator. Following enzymatic digestion, tissues were resuspended in DMEM containing 10% heat-inactivated, sterile-filtered fetal bovine serum (hiFCS; PeloBiotech, Cat. PB-FCS-EU-0500), 100 U/mL penicillin, 100 μg/mL streptomycin (Sigma-Aldrich, Cat. P4333), 2.5 μg/mL amphotericin B (Sigma-Aldrich, Cat. A2942), and 2 mg/ml DNase I. The cell suspension was gently homogenized, transferred to T75 flasks, and cultured at 37 °C in 5% CO_2_. Medium was refreshed after 7 days, followed by an additional 7-day incubation to allow mixed glial cultures to reach confluence. On day 14, adherent microglial cells were separated from astrocytes by incubation with 2 mM PBS-EDTA and gentle shaking.

Harvested microglia were then seeded into appropriate culture plates for downstream assays. Purity of the isolated microglia ranged from 94–99%, as verified by Iba1 immunostaining (**Supplementary Figure 2A**), consistent with yields reported in previous studies (37, 38).

### 2.3 Isolation of gut microbiota-derived small EVs

**Figure 1:**
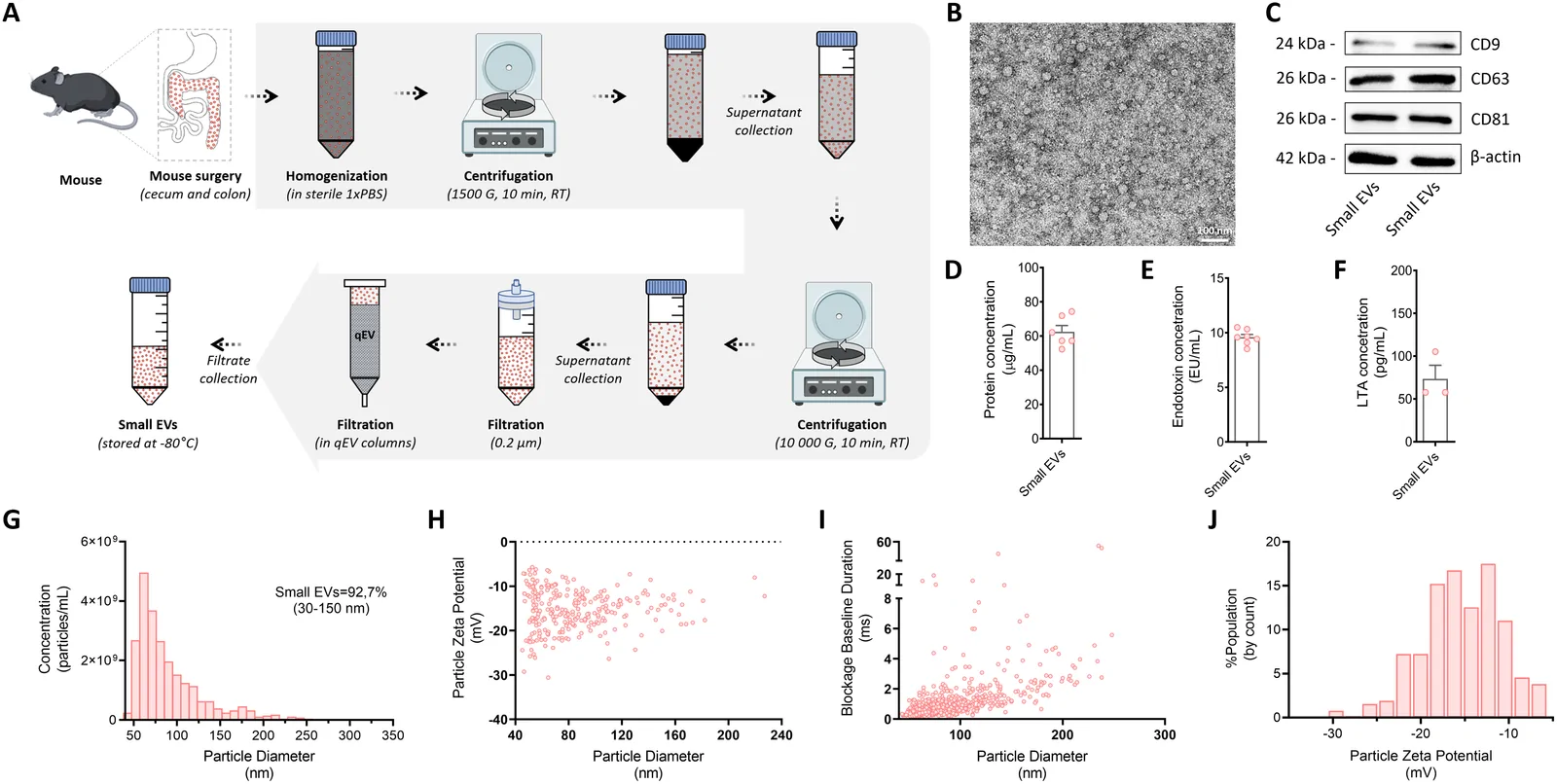
Schematic overview of murine stool–derived small extracellular vesicle (sEV) isolation, purification, and characterization. **(A)** Murine stool collected from the cecum and colon was homogenized in DPBS and subjected to sequential centrifugation to remove debris, followed by 0.2 µm filtration. Small EVs were subsequently purified by size-exclusion chromatography (qEV columns), and vesicle-containing fractions were collected and stored at −80 °C for downstream analyses. Isolated small EVs were characterized by **(B)** TEM for their morphology and by **(G–J)** TRPS/qNano for particle size distribution, concentration, zeta potential, translocation kinetics and relative abundance of vesicles. **(C)** Exosome glycoprotein markers (CD9, CD63, CD81) and protein expression of β-actin were quantified by SDS western-blotting, whereas the **(D)** total protein content was quantified using Pierce™ 660 nm Protein Assay Kit. Bacterial-associated components such as **(E)** endotoxin and **(F)** lipoteichoic acid (LTA) were quantified using commercially available kits. Data were presented as scatter dot plots, mean ± SEM (repeated measurements).

Stool samples were obtained by pooling material from the cecum and colon of adult wild-type C57BL/6J mice aged 3–6 months. Sample collection and downstream processing were performed in accordance with technical recommendations provided by Izon Science Ltd., as schematically summarized in **Figure 1A**. In accordance with Minimal Information for Studies of Extracellular Vesicles (MISEV) 2018/2023 recommendations (39, 40), vesicles isolated in this study are operationally designated as small extracellular vesicles (EVs) based on their size range and separation methodology, without inference of their cellular biogenesis.

Briefly, freshly collected fecal material was diluted in sterile Dulbecco’s phosphate-buffered saline (DPBS; Sigma-Aldrich, Cat. D8537) and mechanically homogenized to achieve a uniform suspension. Particulate debris and larger contaminants were removed by sequential centrifugation steps at 1,500 × g for 10 min, followed by centrifugation of the recovered supernatant at 10,000 × g for 10 min at room temperature. The clarified supernatant was subsequently passed through a 0.2 µm non-pyrogenic filter (Filtropur S, Sarstedt; Cat. 83.1826.001) to eliminate remaining large particles and microbial contaminants.

Size-exclusion chromatography was then performed using qEV SP5 columns (Izon Science Ltd.), with careful handling to maintain a continuous flow and avoid column disturbance, as flow irregularities can compromise the resolution of small extracellular vesicle separation. Purified small EV fractions were immediately stored at −80 °C until further analysis. Total protein content of vesicle preparations was determined using the Pierce™ 660 nm Protein Assay, and protein concentration was used as the dosing unit for subsequent cell stimulation experiments in this study.

For comparative experiments, small EVs were additionally isolated from the murine colorectal epithelial cell line CMT-93 (Cat. 305761, Cytion) and the Gram-positive bacterium *Lacticaseibacillus rhamnosus* PA11 (Cat. DSM 111733, Leibniz Institute DSMZ-German Collection of Microorganisms and Cell Cultures GmbH, Germany) as described above. Outer membrane vesicles (OMVs) derived from *Escherichia coli* were obtained commercially (Cat. tlrl-omv-1, InvivoGen).

### 2.4 Characterization of small EVs by tunable resistive pulse sensing

In accordance with the MISEV2018/2023 guidelines, small EVs were characterized by tunable resistive pulse sensing (TRPS) using the qNano system (Izon Science) to assess particle size distribution, concentration, surface charge (zeta potential), translocation kinetics (blockage magnitude duration), and relative abundance of vesicles within defined size intervals. Measurements were performed using NP100 nanopores, which allow detection of particles approximately 50–300 nm in diameter, in accordance with the manufacturer’s specifications. Size and charge calibration was performed using CPC100 reference particles (modal diameter: 100 nm; Izon Science). Before analysis, vesicle preparations were diluted 1:1 in 2× PBS containing 0.1% Tween 20, following the supplier’s recommendations to ensure optimal pore stability and particle translocation.

Recordings were acquired at a pore stretch of 44.29 mm with an applied voltage of 0.44 V, maintaining a baseline current of 100 ± 10 nA. For each sample, a minimum of 500 individual particle events was collected. To minimize aggregation and ensure sample homogeneity, all preparations were vortex-mixed for 30 s and subjected to brief sonication (2 min) immediately prior to measurement. Surface charge values were calculated using a four-point calibration curve generated with CPC100 standards, as previously described by the manufacturer (41, 42).

### 2.5 Transmission electron microscopy

Purified small extracellular vesicles were examined by transmission electron microscopy (TEM). For sample preparation, vesicle-containing fractions were applied to glow-discharged, carbon-coated Formvar grids (2 nm carbon layer) by placing the grid onto a 20 µL droplet of sample for 10 s to allow particle adsorption. Excess material was removed by briefly washing the grids three times on droplets of ultrapure water. Negative contrast was achieved by staining with 3% (w/v) aqueous uranyl acetate, followed by air drying.

Imaging was performed using a JEOL JEM-1400 transmission electron microscope (JEOL Ltd.) equipped with a bottom-mounted high-sensitivity 4K CMOS camera (TemCam F416, TVIPS) (43).

### 2.6 Quantification of endotoxin and lipoteichoic acid

The levels of Gram-negative endotoxin and Gram-positive lipoteichoic acid (LTA) in isolated small EVs were quantified using commercially available ELISA kits. Endotoxin content was measured with the Pierce LAL Chromogenic Endotoxin Quantitation Kit (Cat. 88282, Thermo Fisher Scientific) following the manufacturer’s protocol. Absorbance was recorded at 405 nm using a Bio-Rad iMark microplate reader, and endotoxin concentrations were calculated from a standard curve and expressed as endotoxin units per milliliter (EU/mL).

LTA concentrations were determined using a Mouse LTA ELISA Kit (Cat. MBS261639, MyBioSource). Samples and standards were processed according to the supplier’s instructions, with absorbance read at 450 nm on the same microplate reader. LTA levels were interpolated from the standard curve and reported as picograms per milliliter (pg/mL).

### 2.7 Microglia cell stimulation

**Figure 2:**
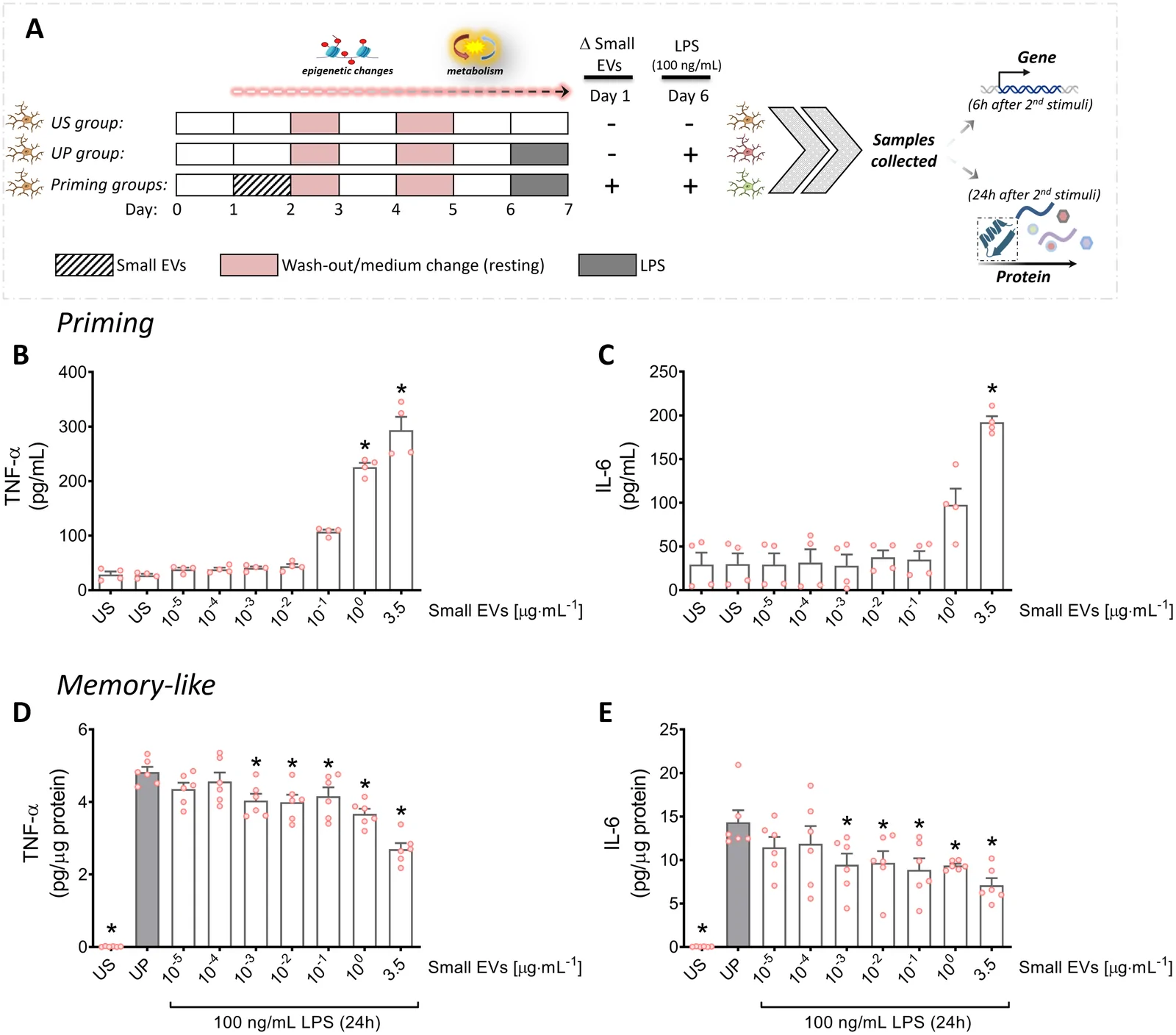
Two-step stimulation protocol and cytokine responses following priming with increasing doses of small EVs. **(A)** Schematic representation of the two-step experimental design used to assess microglial memory-like responses. Primary microglia were primed on day 1 with escalating concentrations of small EVs for 24 h (day 1–2), followed by washout and replacement with fresh medium. A second medium change was performed on day 4 to maintain culture conditions. On day 6, cells were restimulated with LPS (100 ng/mL). Transcriptomic analyses were performed 6 h after LPS stimulation, while protein and cytokine measurements were collected 24 h after LPS stimulation. Control conditions included unstimulated cells maintained in medium alone and unprimed cells receiving LPS only on day 6 without prior EV exposure. Cytokine levels of TNF-α and IL-6 in culture supernatants measured 24h after the [**(B, C)**; n = 4] single stimulation (priming) with increasing concentrations of small EVs doses as well as after [**(D, E)**; n = 6] LPS restimulation (memory-like reactions) were quantified by ELISA and normalized to total protein content. Data were presented as scatter dot plots, mean ± SEM, Kruskal-Wallis [Dunn’s *post-hoc*; **(B, C)**] and 1-Way ANOVA [Holm-Sidak *post-hoc*; **(D, E)**], *p<0.05, * indicating statistical significance compared to **(B, C)** unstimulated or **(D, E)** unprimed control.

To investigate the induction of memory-like responses, primary microglial cells (75,000 cells per well) were treated according to the stimulation scheme illustrated in **Figure 2A** and based on previously established protocols (36, 38, 44). Cells underwent a two-step stimulation procedure with an initial priming step using increasing protein concentrations of isolated small EVs (first challenge: 10 pg/mL−3.5 µg/ml) for 24h on day 1. Following priming, cultures were washed, and fresh medium was added; a second medium replacement was performed on day 4 to maintain optimal cell viability and nutrient levels. On day 6, microglial cells were restimulated with a fixed dose of 100 ng/mL LPS (*Escherichia coli* serotype 055:B5, Cat. tlrl-pb5lps, InvivoGen) and incubated at 37 °C and 5% CO_2_. Samples were harvested 6 h post-restimulation for transcriptomic analysis and 24 h post-restimulation for cytokine and protein measurements. Control conditions included unstimulated (US) cells maintained in medium alone, and unprimed (UP) cells receiving only LPS on day 6 without prior small EV exposure. This design enabled distinction between baseline LPS responses and priming-dependent memory-like effects.

### 2.8 Antibodies

Primary antibodies were obtained from Cell Signaling Technology unless otherwise stated: MyD88 (1:1000, rabbit, Cat. 4283), phospho-p38 MAPK (1:1000, rabbit, Cat. 9211), p38 MAPK (1:1000, rabbit, Cat. 9212), phospho-p44/42 MAPK (Erk1/2) (1:2000, mouse, Cat. 9106), p44/42 MAPK (Erk1/2) (1:1000, mouse, Cat. 9107), phospho-NF-κB p65 (1:1000, rabbit, Cat. 3033), TLR2 (1:1000, rabbit, Cat. 13744), Tri-Methyl-Histone H3 (Lys4) (H3K4me3, 1:1000, rabbit, Cat. 9751), Tri-Methyl-Histone H3 (Lys9) (H3K9me3, 1:1000, rabbit, Cat. 13969), Acetyl-Histone H3 (Lys27) (H3K27ac, 1:1000, rabbit, Cat. 8173), and H3 (1:2000, rabbit, Cat. 4499). Antibodies against TLR4 (1:200, mouse, Cat. sc-293072), CD9 (1:200, mouse, Cat. sc-13118), CD63 (1:200, mouse, Cat. sc-5275), CD81 (1:100, mouse, Cat. sc-5275) were purchased from Santa Cruz Biotechnology, whereas anti-L-Lactyl-Histone H3 (Lys18) (H3K18la, 1:500, rabbit, Cat. PTM-1406RM) was sourced from PTM BIO. β-actin antibody (1:1000, mouse, Cat. 3700, Cell Signaling) was used as a loading control. Horseradish peroxidase (HRP)-conjugated secondary antibodies, anti-rabbit (1:10,000; Cat. 111-035-144) and anti-mouse (1:10,000; Cat. 115-035-166), were obtained from Dianova.

Normalization to total histone H3 was not feasible due to weak and inconsistent H3 signals under the RIPA-based extraction conditions used in this study. Therefore, histone modification levels were normalized to β-actin, which served as a stable loading control across all samples. All samples were processed under identical conditions to ensure reliable comparative analyses.

### 2.9 Total protein quantification

Total protein content in cell lysates was quantified using the Pierce™ 660 nm Protein Assay (Cat. 22662, Thermo Fisher Scientific) in combination with the Ionic Detergent Compatibility Reagent (IDCR; Cat. 22663, Thermo Fisher Scientific) to ensure reliable measurement in detergent-containing samples. Protein standards, samples, and blanks were dispensed in duplicate into 96-well plates (10 µL per well), followed by the addition of the supplemented assay reagent according to the manufacturer’s protocol. After incubation, absorbance was recorded at 660 nm using a Bio-Rad iMark microplate reader, and total protein concentrations were calculated based on a standard curve.

### 2.10 SDS–PAGE and immunoblotting

Cells were lysed on ice using ice-cold radioimmunoprecipitation assay (RIPA) buffer composed of 50 mM Tris–HCl (pH 8.0), 150 mM NaCl, 1% NP-40, 0.5% sodium deoxycholate, and 0.1% SDS. Lysis buffer was freshly supplemented with protease and phosphatase inhibitor tablets (Cat. A32959, Thermo Fisher Scientific). Cell suspensions were briefly vortexed, incubated on ice to facilitate complete lysis, and clarified by centrifugation at 12,000 × g for 30 min at 4 °C. The resulting supernatants were collected and stored at −80 °C until analysis. For immunoblotting, protein lysates were combined with 5× Laemmli sample buffer and denatured by heating at 95 °C for 5 min. Equal amounts of protein were separated on 10% SDS–polyacrylamide gels and transferred onto 0.45 μm polyvinylidene fluoride (PVDF) membranes. Membranes were blocked for 30–45 min at room temperature in Tris-buffered saline containing 0.1% Tween-20 (TBST) and 1% bovine serum albumin to reduce nonspecific binding. Primary antibody incubations were performed overnight at 4 °C, followed by extensive washing with TBST. Membranes were then incubated with horseradish peroxidase–conjugated secondary antibodies for 1 h at room temperature.

Protein signals were detected using enhanced chemiluminescence reagents (WesternBright Sirius Chemiluminescent Detection Kit, Cat. 541020, Biozym) and visualized with a Bio-Rad ChemiDoc™ XRS+ imaging system. Band intensities were quantified using Image Lab software (version 6.0.1; Bio-Rad Laboratories).

### 2.11 Enzyme-linked immunosorbent assay

Secretion of cytokines and chemokines into cell culture supernatants was quantified by enzyme-linked immunosorbent assay (ELISA). Supernatants were harvested 24 h after the secondary LPS stimulation, cleared by centrifugation at 10,000 × g for 5 min at 4 °C to remove residual cellular material, and stored at −80 °C until analysis. Concentrations of TNF-α (Cat. 430901), IL-6 (Cat. 431301), IL-10 (Cat. 431411), IL-1β (Cat. 432601), and MCP-1 (Cat. 432701) were determined using commercially available ELISA kits (BioLegend) according to the manufacturer’s instructions and as previously reported (45, 46). Absorbance was measured at 450 nm with wavelength correction at 570 nm using an iMark microplate reader (Bio-Rad Laboratories). Cytokine and chemokine levels obtained after the secondary challenge were normalized to the total protein content of the corresponding samples and are presented as picograms per microgram of total protein.

### 2.12 Quantification of reactive oxygen species

Primary microglial cells were plated at a density of 25,000 cells per well in black, clear-bottom 96-well plates and stimulated according to the experimental scheme described above. ROS generation was quantified using a fluorometric DCFDA-based assay (Cellular ROS Assay Kit, Cat. ab113851, Abcam). Prior to fluorescence measurement, cells were incubated with 20 µM 2′,7′-dichlorodihydrofluorescein diacetate (DCFDA) for 45 min at 37 °C in the absence of light. Excess dye was removed, and cells were washed and resuspended in freshly prepared 1× assay buffer provided with the kit. Following cellular uptake, DCFDA will be enzymatically deacetylated by intracellular esterases and subsequently oxidized by reactive oxygen species to the fluorescent product 2′,7′-dichlorofluorescein (DCF). Fluorescence signals were acquired using a Fluoroskan FL microplate reader (Thermo Fisher Scientific) at excitation and emission wavelengths of 485 nm and 535 nm, respectively.

### 2.13 Transwell-based transmigration assay

The migratory capacity of primary microglial cells *in vitro* was evaluated using a transwell assay as previously described (47, 48). Microglia were resuspended at 1.5 × 10^5^ cells in 100 μL of 0.5% FCS-containing medium and carefully added to the upper chamber of 24-well transwell inserts with 8 μm pores (Greiner Bio-One, Cat. 662638). The lower chamber was filled with 600 μL of migration medium supplemented with 20% heat-inactivated FCS to serve as a chemoattractant. Cells were incubated for 4 h at 37 °C under 5% CO_2_ to allow migration through the membrane. After the incubation, non-migrated cells on the upper surface of the membrane were gently removed with a cotton swab. Migrated cells on the lower side were fixed in 4% sterile-filtered paraformaldehyde for 10–15 min at room temperature. Following fixation, membranes were stained with 1 μg/mL 4′,6-diamidino-2-phenylindole (DAPI) for 10 min in the dark and washed three times with PBS. The inserts were air-dried and mounted onto microscope slides.

Fluorescent images were acquired using a Leica fluorescence microscope (DFC450 Camera, Leica Microsystems, version 4.9.0) with a 10× objective. The number of transmigrated cells was determined by counting DAPI-stained nuclei in five randomly selected fields per membrane and expressed as the absolute number of migrated cells.

### 2.14 Microglial phagocytic activity

The phagocytic capacity of primary microglial cells *in vitro* was assessed using the CytoSelect™ 96-well Phagocytosis Assay with Zymosan particles (*Saccharomyces cerevisiae*; Cat. CBA-224, Cell Biolabs) following the manufacturer’s protocol. Microglia were plated at 1 × 10^5^ cells per well in 96-well plates and treated according to the experimental conditions described above. A suspension of Zymosan particles (10 µL of 5 × 10^8^ particles/mL) was added to each well, and cells were incubated for 2 h at 37 °C in a humidified 5% CO_2_ atmosphere to allow particle internalization.

After incubation, non-internalized particles were removed by repeated washes with serum-free DMEM, and cells were fixed according to the kit instructions. Following fixation, cells were blocked, permeabilized briefly, and washed with PBS. Internalized Zymosan particles were quantified by measuring absorbance at 405 nm using an iMark microplate reader (Bio-Rad Laboratories). Phagocytic activity was reported as the optical density (OD) corresponding to the amount of internalized substrate.

### 2.15 Quantification of lactate production

Lactate levels in culture supernatants were quantified using the L-Lactate Assay Kit (Cat. K607, BioVision Inc) following the manufacturer’s instructions. A 10 nmol/well L-lactate standard was diluted in assay buffer, followed by a two-fold serial dilution to generate a standard curve, with assay buffer alone as the blank. Standards, samples, and blanks (50 µL/well) were loaded in duplicate into a 96-well plate. Subsequently, 50 µL of freshly prepared reaction mix (2 µL lactate enzyme mix, 2 µL probe, 46 µL assay buffer) was added to each well and mixed gently. Plates were incubated for 30 min at room temperature protected from light, and absorbance was measured at 570 nm using an iMark microplate reader (Bio-Rad Laboratories). Lactate concentrations were calculated from the standard curve and expressed as micromolar (µM) in the final reaction volume.

### 2.16 cDNA synthesis and quantitative real-time PCR

Six hours after the secondary challenge, total RNA was isolated from primary microglial cells using TRIzol™ Reagent (Cat. 15596018, Invitrogen), as previously described (45, 48). RNA yield and purity were assessed with a DS11 FX+ spectrophotometer (DeNovix). Reverse transcription was performed with the High-Capacity cDNA Reverse Transcription Kit (Cat. 4368814, Applied Biosystems) to generate cDNA. Quantitative real-time PCR was carried out on a StepOnePlus instrument (Applied Biosystems) to quantify transcript levels of the indicated target genes.

The following primer pairs were used: IL-1β (F: GGCAGGCAGTATCACTCATT; R: AAGGTGCTCATGTCCTCAT), IL-10 (F: ACCAGCTGGACAACATACTGC; R: TCACTCTTCACCTGCTCCACT), PFK-1 (F: TGACATGACCATTGGCACAG; R: TCTTGCTACTCAGGATTCGG), HK-2 (F: ATTGTCCAGTGCATCGCGGA; R: AGGTCAAACTCCTCTCGCCG), PFKFB3 (F: GGAGAGGTCAGAGGATGCAAA; R: GCTGTTGATGCGAGGCTTTT), CD32 (F: AATCCTGCCGTTCCTACTGATC; R: GTGTCACCGTGTCTTCCTTGAG), CD11a (F: AGATCGAGTCCGGACCCACAG; R: GGCAGTGATAGAGGCCTCCCG), NFκB-p65 (F: CTTCCTCAGCCATGGTACCTCT; R: CAAGTCTTCATCAGCATCAAACTG), and GAPDH (F: CATGGCCTTCCGTGTTTCCTA; R: CCTGCTTCACCACCTTCTTGAT). GAPDH served as the reference gene, and relative expression was calculated using the comparative Ct method (2^-ΔΔCT^).

### 2.17 Assessment of cell viability and cytotoxicity

To assess primary microglial viability and cytotoxicity, cells were plated at 5,000 cells per well and stimulated according to the experimental scheme described above. 24h after the LPS challenge, metabolic activity was quantified by a 3-[4,5-dimethylthiazol-2-yl]-2,5 diphenyl tetrazolium bromide (MTT) assay: 10 µL of MTT labeling solution (5 mg/mL) was added per well and incubated for 4 h at 37 °C. Formazan crystals were solubilized by adding 100 µL of solubilization buffer (0.01 M HCl, 20% SDS), followed by overnight incubation. In parallel, cytotoxicity was determined using a colorimetric Cell Cytotoxicity Assay Kit (Cat. ab112118, Abcam). Briefly, 20 µL of the provided assay reagent was added to each well and incubated for 4 h at 37 °C in 5% CO_2_ protected from light.

Absorbance for each assay was recorded at 570 nm using a Bio-Rad iMark microplate reader. Data were expressed as relative viability or cytotoxicity, normalized to unstimulated controls set to 100% (**Supplementary Figures 2B, C**).

### 2.18 Statistical analysis

Figures and statistical analyses were performed using GraphPad Prism (v8.0.2; GraphPad Software) and SigmaPlot (v12.0; Systat Software GmbH). Data distribution was evaluated with the Shapiro–Wilk normality test. For datasets that did not meet normality assumptions, group comparisons were conducted using the Kruskal–Wallis test followed by Dunn’s multiple-comparisons procedure. For normally distributed data, differences among groups were assessed by one-way analysis of variance (ANOVA) with Holm–Sidak correction for *post hoc* testing. Results are presented as mean + SEM, and statistical significance was defined as p < 0.05.

## 3 Results

### 3.1 Characterization of murine gut-derived small EVs

Gut-derived small EVs were obtained from adult mice following the workflow outlined in **Figure 1A**. TEM confirmed the presence of predominantly spherical vesicles enclosed with a diameter below 100 nm (**Figure 1C**). Protein expression of transmembrane proteins (CD9, CD63 and CD81) as key members of tetraspanin family for identifying small EVs as well as β-actin were confirmed by immunoblot detection (**Figure 1C**). Total protein content was quantified and subsequently used to standardize small EV dosing in all downstream experiments (**Figure 1D**). Consistent with their microbial origin, small EV samples contained detectable levels of endotoxin and LTA (**Figures 1E, F**).

TRPS/qNano system was used to assess vesicle size distribution and particle abundance, revealing a preparation highly enriched in small EVs (>92%) with consistent particle characteristics (**Figure 1G**). Measurements of surface charge indicated stable electrostatic properties, with zeta potential values below −10 mV across preparations (**Figure 1H**). Blockade duration was consistently below 10 milliseconds for most events, indicating rapid and uniform translocation through the nanopore (**Figure 1I**). Population analysis based on particle counts showed that the predominant fraction of vesicles comprised the major population (**Figure 1J**). Independent zeta potential measurements demonstrated that this dominant population exhibited a stable negative surface charge, with values largely ranging between −20 and −10 mV, consistent with electrostatically stable vesicles.

### 3.2 Concentration-dependent modulation of trained innate immune tolerance responses by small EVs

Primary murine microglial cells exposed to gut-derived small EVs exhibited concentration-dependent effects on inflammatory responses. At concentrations starting from 1 μg/mL, small EV priming significantly increased the production of prototypical pro-inflammatory cytokines TNF-α and IL-6 (**Figures 2B, C**; p < 0.05). Following a secondary inflammatory challenge with LPS according to the two-step stimulation protocol, higher concentrations of small EVs induced memory-like responses characterized by reduced TNF-α and IL-6 levels, indicative of *in vitro* trained innate immune tolerance (**Figures 2D, E**). To ensure that these effects were not confounded by differences in cell survival, microglial viability and cytotoxicity were assessed using MTT and cytotoxicity assays, confirming no significant differences between treatment groups (**Supplementary Figures 2B, C**).

To better define the origin of the immunomodulatory effects observed with gut-derived small EVs, vesicles from host and bacterial sources were examined separately. Small EVs were isolated from murine colorectal epithelial CMT-93 cells and the Gram-positive bacterium *Lacticaseibacillus rhamnosus* as described above, whereas OMVs from the Gram-negative bacterium *Escherichia coli* were obtained commercially. Exposure of primary microglia to colorectal-derived small EVs (50 µg/mL) did not alter TNF-α or IL-6 production following a single stimulation. In contrast, OMVs from *E. coli* (10 µg/mL) and cytoplasmic membrane vesicles (CMVs) from *L. rhamnosus* (25 µg/mL) induced a significant increase in the release of both cytokines (**Supplementary Figures 1A, B**; p < 0.05). Interestingly, after secondary LPS stimulation, colorectal-derived small EVs enhanced TNF-α and IL-6 production, whereas *E. coli* OMVs and *L. rhamnosus* CMVs reduced cytokine levels compared with unprimed controls (**Supplementary Figures 1C, D**). Together, these findings suggest that the gut-derived small EV preparations used throughout this study predominantly reflect bacterial vesicle-mediated immunomodulatory activity and support a role for bacterial EVs as key drivers of trained innate immune tolerance in microglia.

**Figure 3:**
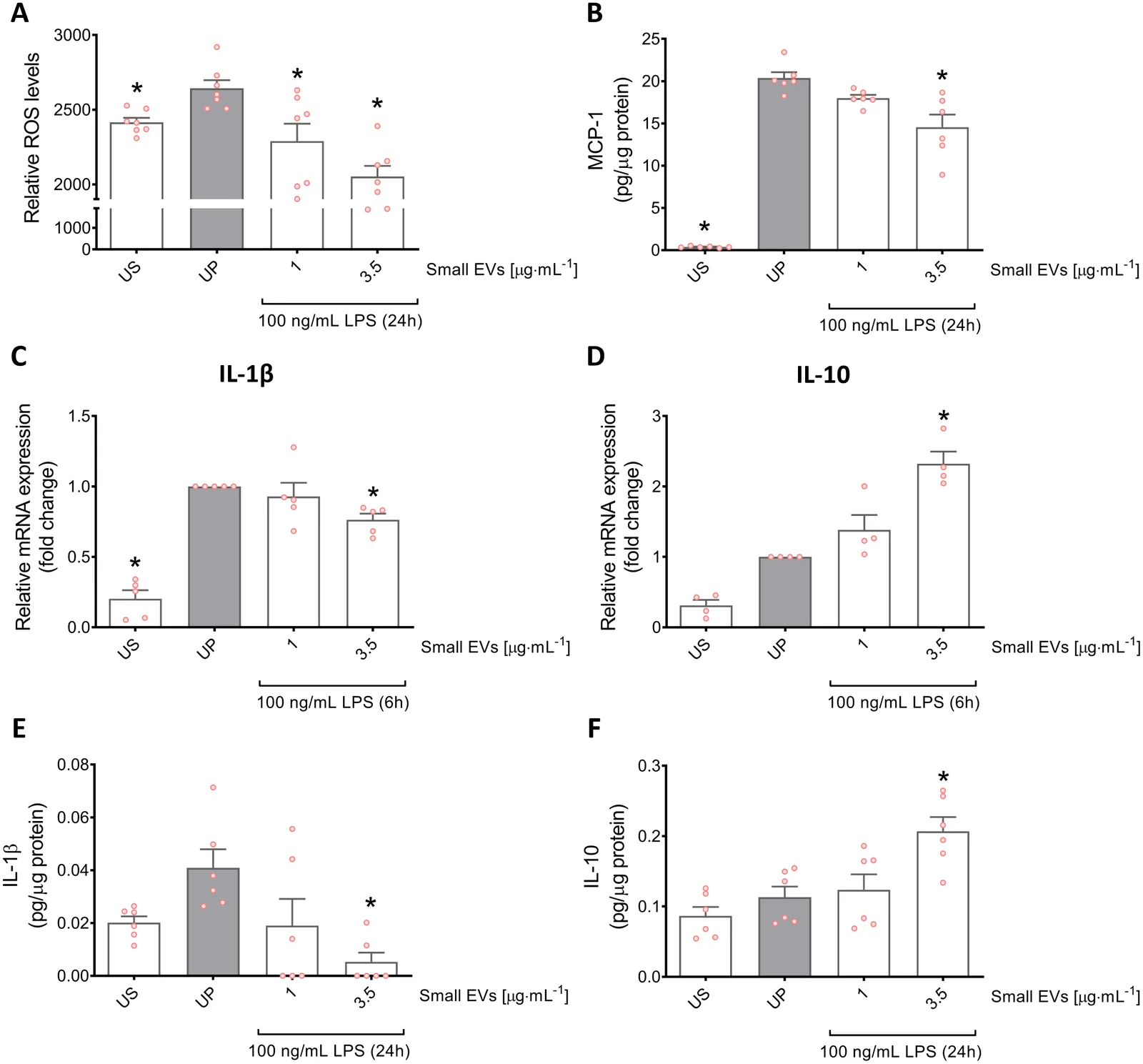
Small EVs drive innate immune tolerance in primary microglia under inflammatory challenge. Primary microglial cultures were analyzed using a two-step stimulation paradigm. Cells were initially exposed to small extracellular vesicles (small EVs; 1 or 3.5 µg/mL) for 24 h to induce priming, after which vesicles were removed and cultures were allowed to recover following medium replacement. On day 7, microglia were re-stimulated with LPS (100 ng/mL). Reactive oxygen species (ROS) production [**(A)** n = 7] was quantified using a DCFDA-based fluorometric assay. Supernatants collected 24 h after LPS re-stimulation were analyzed for MCP-1 [**(B)** n = 6], IL-1β [**(E)** n = 6], and IL-10 [**(F)** n = 6] by ELISA and normalized to total protein content. Total RNA was isolated 6 h after LPS exposure, and mRNA expression of IL-1β [**(C)** n = 5] and IL-10 [**(D)** n = 4] was assessed by quantitative real-time PCR. Data were presented as scatter dot plots, mean ± SEM, 1-Way ANOVA [Holm-Sidak *post-hoc*; **(A, C, F)**] and Kruskal-Wallis [Dunn’s *post-hoc*; **(B, D, E)**], *p<0.05, * indicating statistical significance compared to unprimed control.

To further explore the capacity of gut-derived small EVs to promote trained innate immune tolerance, we focused on the two higher small EV concentrations that had elicited both enhanced inflammatory responses upon single stimulation and tolerance upon two-step stimulation. At these concentrations, we expanded the panel of inflammatory markers and observed that small EV-primed microglia exhibited reduced production of ROS, MCP-1, and IL-1β following LPS re-challenge (**Figures 3A–C, E**; p < 0.05). Concurrently, these memory-like responses were associated with an anti-inflammatory phenotype, as evidenced by elevated IL-10 gene expression and cytokine secretion (**Figures 3D, F**; p < 0.05).

### 3.3 Small EV priming suppresses TLR-dependent inflammatory signaling in microglia

**Figure 4:**
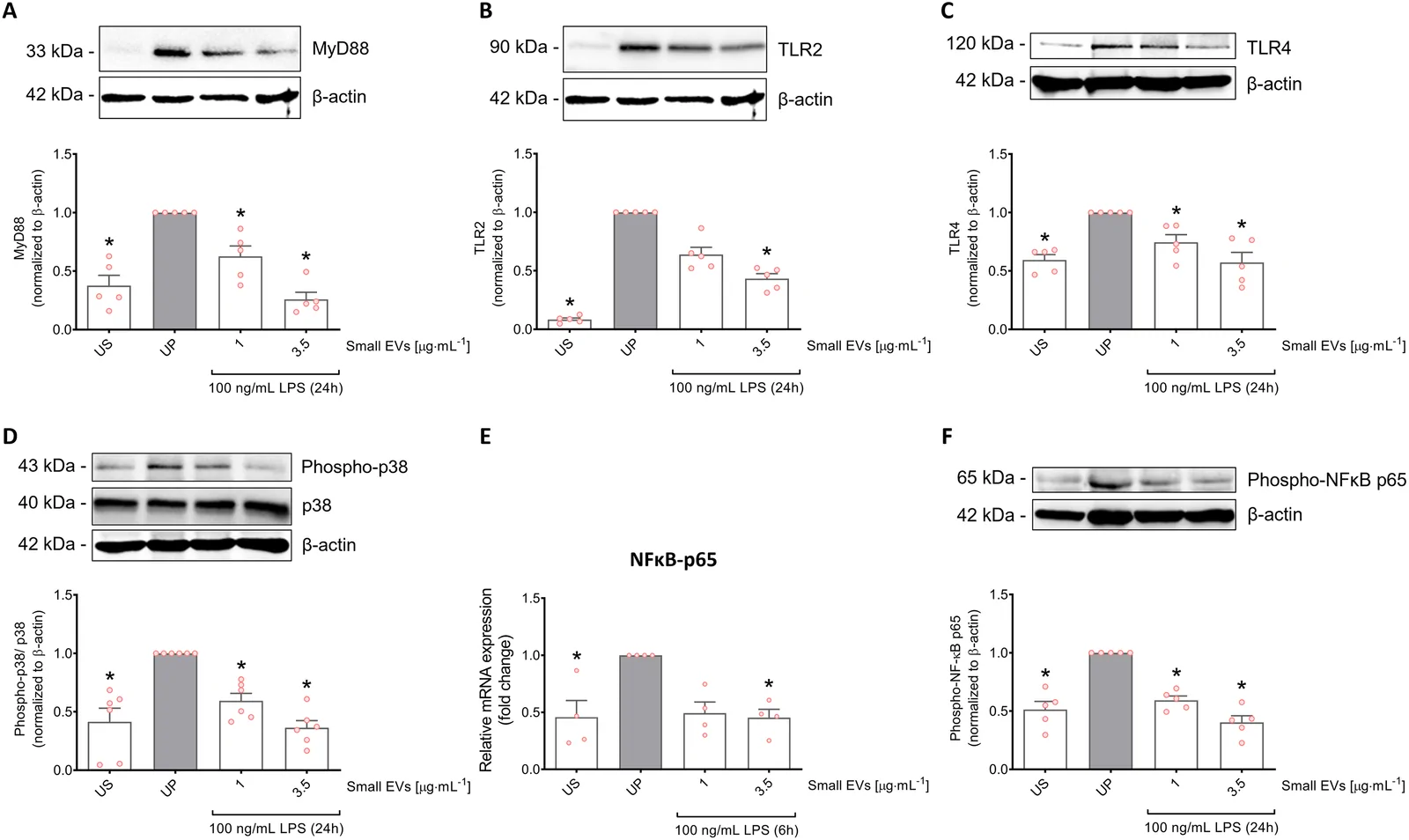
Small EVs attenuate microglial pro-inflammatory signaling by modulating TLR/MyD88 and MAPK–NF-κB pathways. Primary microglial cultures were analyzed using a two-step stimulation paradigm. Cells were initially exposed to small extracellular vesicles (small EVs; 1 or 3.5 µg/mL) for 24 h to induce priming, after which vesicles were removed and cultures were allowed to recover following medium replacement. On day 7, microglia were re-stimulated with LPS (100 ng/mL). Protein lysates collected 24 h post-treatment were analyzed for protein expression of [**(A)**, n = 5] MyD88, [**(B)**, n = 5] TLR2, [**(C)**, n = 5] TLR4, [**(D)**, n = 6] phospho-p38 and [**(F)**, n = 5] phospho-NFκB-p65 by immunoblotting and quantified (unprimed cells were set to 1.0). Total RNA was isolated 6 h following LPS exposure, and transcript levels of [**(E)**, n = 4] NFκB-p65 were determined by quantitative real-time PCR. Data were presented as scatter dot plots, mean ± SEM, 1-Way ANOVA [Holm-Sidak *post-hoc*; **(A, C, D, F)**] and Kruskal-Wallis [Dunn’s *post-hoc*; **(B, E)**], *p<0.05, * indicating statistical significance compared to unprimed control. Panels A (MyD88) and B (TLR2) were detected on the same membrane and therefore share the same β-actin loading control.

To elucidate the inflammatory signaling pathways affected by small EV priming and the downstream proteins involved in memory-like responses with reduced pro-inflammatory output, we focused on TLR-mediated mechanisms. Given the presence of endotoxin and LTA in the small EV preparations, we examined TLR2 and TLR4, which recognize these microbial components, along with their adaptor protein myeloid differentiation primary response protein 88 (MyD88). Immunoblot analysis revealed that MyD88, TLR2, and TLR4 protein levels were significantly decreased in microglia primed with 1 or 3.5 μg/mL small EVs compared with unprimed controls (**Figures 4A–C**; p < 0.05).

Because MAPKs are key downstream mediators in memory-like innate immune responses, we next assessed p38 MAPK activation. Phosphorylation of p38 was markedly reduced in small EV-primed microglia, corresponding with decreased production of inflammatory mediators (**Figure 4D**). TLR signaling and p38 MAPK activation are known to trigger phosphorylation of inhibitor of nuclear factor kappa B (IκB), which facilitates nuclear translocation of NF-κB. Consistent with this pathway, small EV-primed microglia exhibited decreased NF-κB p65 activation, indicating suppressed NF-κB signaling *in vitro* (**Figures 4E, F**).

### 3.4 Metabolic reprogramming accompanies trained innate immune tolerance in microglia

**Figure 5:**
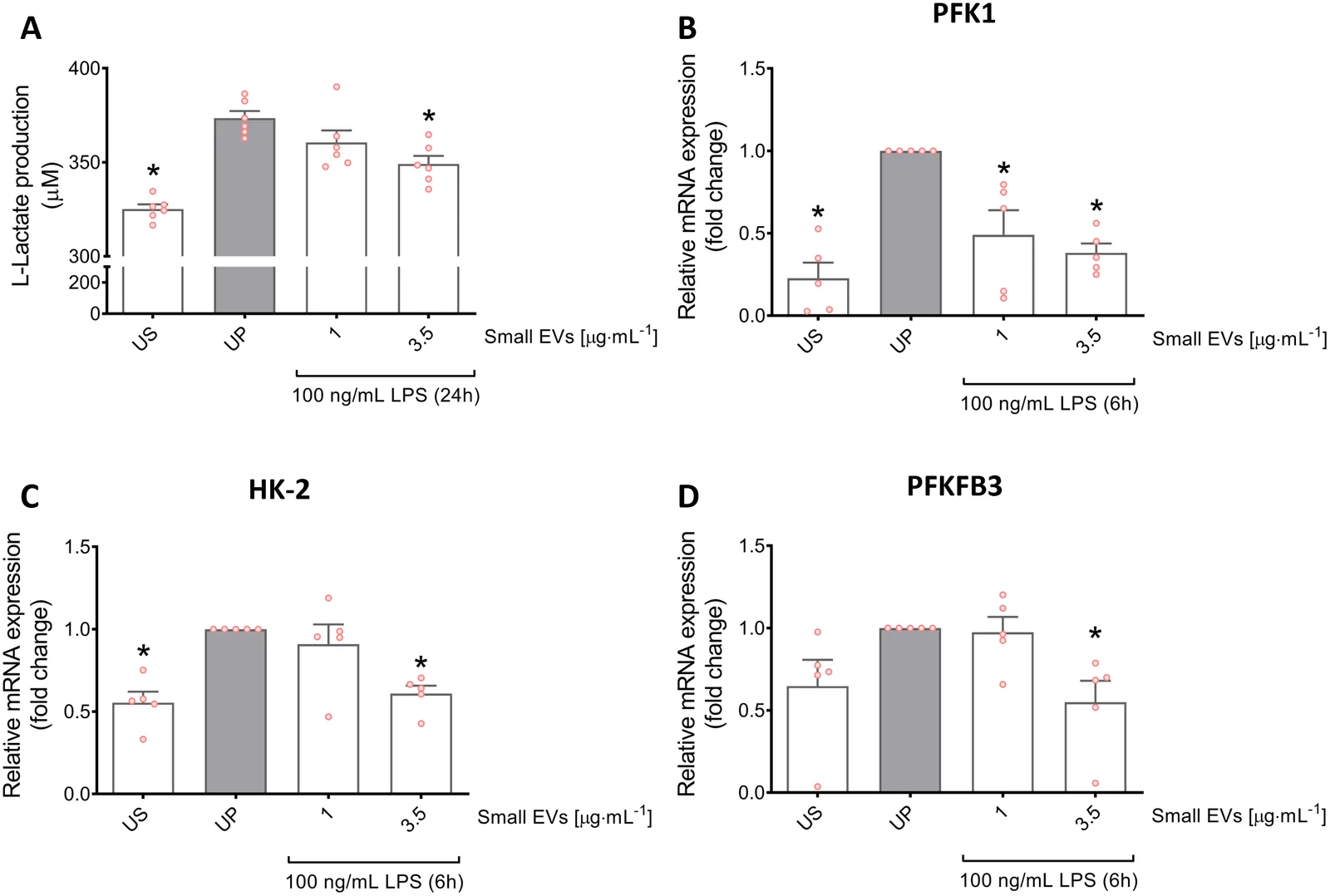
Enhanced glycolytic metabolism accompanies inflammation-tolerant innate immune training in microglia. Primary microglial cultures were analyzed using a two-step stimulation paradigm. Cells were initially exposed to small extracellular vesicles (small EVs; 1 or 3.5 µg/mL) for 24 h to induce priming, after which vesicles were removed and cultures were allowed to recover following medium replacement. On day 7, microglia were re-stimulated with LPS (100 ng/mL). Culture supernatants collected 24 h after LPS exposure were assessed for L-lactate accumulation [**(A)**, n = 6] using a colorimetric assay. Total RNA was isolated 6 h following LPS re-stimulation, and transcript levels of glycolytic enzymes like **(B)** PFK1, **(C)** HK-2, and **(D)** PFKFB3 (n = 5) were quantified by real-time PCR. Data were presented as scatter dot plots, mean ± SEM, 1-Way ANOVA [Holm-Sidak *post-hoc*; **(A, B)**] and Kruskal-Wallis [Dunn’s *post-hoc*; **(C, D)**], *p<0.05, * indicating statistical significance compared to unprimed control.

Emerging evidence highlights a close interplay between metabolic reprogramming and inflammatory regulation in innate immune cells. To investigate whether gut-derived small EVs influence glycolytic metabolism in microglia, we quantified L-lactate production as a readout of glycolytic activity (**Figure 5A**). Priming with higher concentrations of small EVs (3.5 μg/mL) resulted in a significant (p < 0.05) reduction in lactate levels, suggesting a suppression of glycolytic flux.

To further explore this metabolic reprogramming, we analyzed the expression of key glycolytic enzymes, including phosphofructokinase-1 (PFK1), hexokinase-2 (HK-2), and 6-phosphofructo-2-kinase/fructose-2,6-bisphosphatase-3 (PFKFB3). Microglia primed with 3.5 μg/mL small EVs exhibited reduced expression of all three enzymes, mirroring the decrease in lactate production, while PFK1 expression was also significantly downregulated at 1 μg/mL small EVs (**Figures 5B–D**; p < 0.05). Reduced lactate production and downregulation of key glycolytic enzymes in small EV-primed microglia coincided with lower levels of inflammatory cytokines following LPS re-stimulation.

### 3.5 Epigenetic remodeling accompanies small EV–mediated reprogramming of microglial responses

Given that metabolic reprogramming in innate immune cells is tightly linked to chromatin remodeling, we next examined whether small EV priming is associated with epigenetic alterations in murine microglia. Metabolic intermediates can directly regulate histone-modifying enzymes and thereby shape inflammatory gene expression programs. Post-translational changes such as methylation, acetylation, and lactylation at specific chromatin regions have been reported to play key roles in establishing memory-like features in innate immune cells. To investigate whether small EV priming induces such epigenetic reprogramming in microglia, we analyzed a set of well-characterized histone marks by immunoblotting.

**Figure 6:**
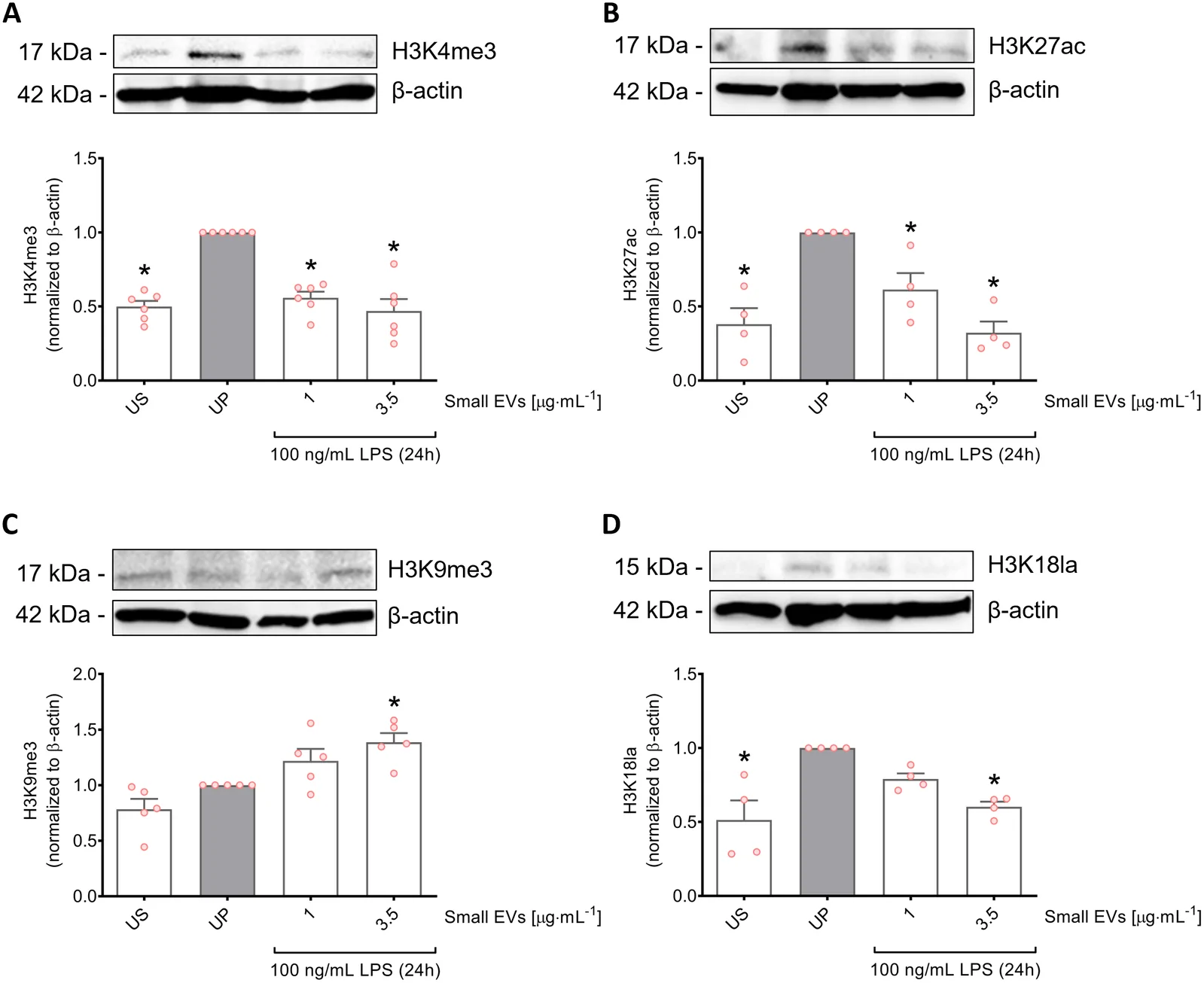
Small EVs reprogram microglial inflammatory and metabolic programs via histone modifications. Primary microglial cultures were analyzed using a two-step stimulation paradigm. Cells were initially exposed to small extracellular vesicles (small EVs; 1 or 3.5 µg/mL) for 24 h to induce priming, after which vesicles were removed and cultures were allowed to recover following medium replacement. On day 7, microglia were re-stimulated with LPS (100 ng/mL). Protein lysates collected 24 h post-stimulation were analyzed by immunoblotting for key histone modifications associated with inflammatory and metabolic regulation, including H3K4me3 [**(A)**, n = 6], H3K27ac [**(B)**, n = 4], H3K9me3 [**(C)**, n = 5], H3K18la [**(D)**, n = 4], and quantified (unprimed cells were set to 1.0). Data were presented as scatter dot plots, mean ± SEM, 1-Way ANOVA [Holm-Sidak *post-hoc*; **(A-D)**], *p<0.05, * indicating statistical significance compared to unprimed control.

Microglia primed with small EVs exhibited a marked reduction in trimethylation of histone H3 at lysine 4 (H3K4me3) and acetylation at lysine 27 (H3K27ac), modifications typically associated with active chromatin and gene transcription (**Figures 6A, B**; p < 0.05). These decreases correlated with the attenuated pro-inflammatory cytokine production observed in these cells. In contrast, trimethylation of histone H3 at lysine 9 (H3K9me3), a modification linked to heterochromatin formation and transcriptional repression, was increased, particularly at the higher small EV concentration (3.5 μg/mL), consistent with suppression of pro-inflammatory gene expression (**Figure 6C**). Additionally, lactylation of histone H3 at lysine 18 (H3K18la), which is associated with glycolytic activity, was reduced, supporting the observed decrease in lactate production during the induction of trained innate immune tolerance (**Figure 6D**; p < 0.05).

### 3.6 Small EV priming enhances microglial phagocytosis and migration

**Figure 7:**
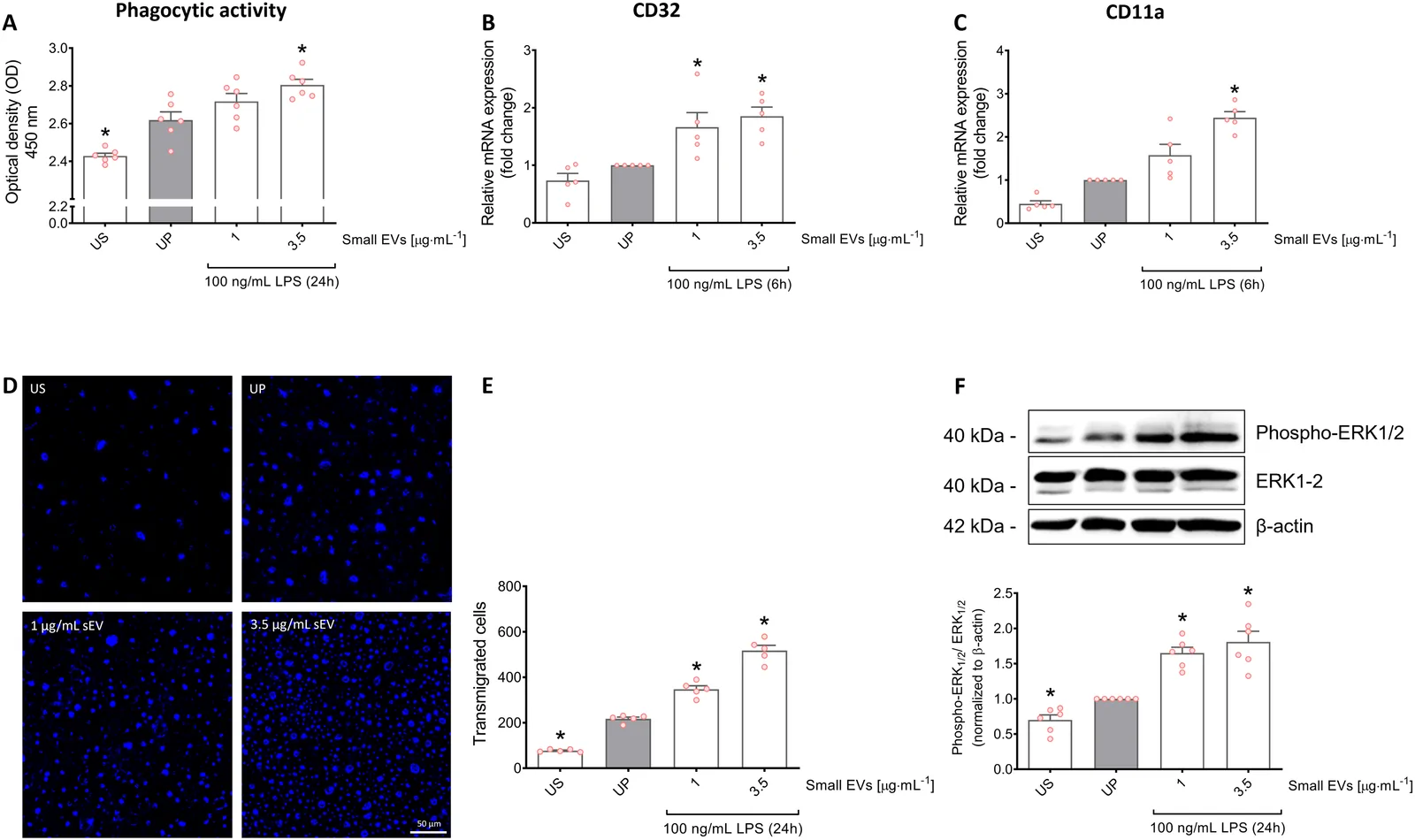
Priming with small EVs enhances microglial phagocytic and migratory functions *in vitro*. Primary microglial cultures were analyzed using a two-step stimulation paradigm. Cells were initially exposed to small extracellular vesicles (small EVs; 1 or 3.5 µg/mL) for 24 h to induce priming, after which vesicles were removed and cultures were allowed to recover following medium replacement. On day 7, microglia were re-stimulated with LPS (100 ng/mL). Phagocytic capacity was assessed 24 h after LPS challenge, using fluorescently labeled zymosan particles derived from *Saccharomyces cerevisiae*, and uptake was quantified as optical density values [**(A)**, n = 6]. Microglial migratory behavior was evaluated using a transwell migration assay, with **(D)** migrated cells visualized by DAPI nuclear staining and [**(E)** n = 5] quantified as absolute cell numbers. Total RNA was isolated 6 h following LPS exposure, and transcript levels of the phagocytosis- and adhesion-related markers **(B)** CD32 and **(C)** CD11a were determined by quantitative real-time PCR (n = 5). Protein lysates collected 24 hours post-treatment were analyzed for protein expression of [**(F)**, n=6] phospho-Erk1/2 by immunoblotting and quantified (unprimed cells were set to 1.0). Data were presented as scatter dot plots, mean ± SEM, 1-Way ANOVA [Holm-Sidak *post-hoc*; **(A, B, E, F)**] and Kruskal-Wallis [Dunn’s *post-hoc*; **(C)**], *p<0.05, * indicating statistical significance compared to unprimed control.

Finally, after delineating the inflammatory, metabolic, and chromatin-associated alterations induced by small EV priming we next determined whether this reprogrammed state is reflected in altered functional responses of primary murine microglia *in vitro*. Phagocytic activity, assessed 24 h after LPS re-stimulation, was significantly enhanced in cells primed with 3.5 μg/mL small EVs (**Figure 7A**; p < 0.05). This functional increase was accompanied by upregulation of CD32, a key phagocytic receptor, at both 1 and 3.5 μg/mL small EV concentrations (**Figure 7B**). Because effective phagocytosis often requires preceding migratory activity, we investigated the impact of small EV priming on microglial migration using a transwell assay. Both 1 and 3.5 μg/mL small EVs significantly increased the number of microglia that migrated through the membrane compared with unprimed controls (**Figures 7D, E**; p < 0.05). Correspondingly, expression of CD11a, an integrin involved in microglial adhesion and migration, was elevated in small EV-primed cells, indicating transcriptional changes supporting enhanced migratory capacity (**Figure 7C**).

Mechanistically, these functional enhancements in phagocytosis and migration were associated with increased activation of ERK 1/2– MAPK signaling. Western blot analysis demonstrated elevated levels of phosphorylated ERK1/2 in small EV-primed microglia (**Figure 7F**; p < 0.05), suggesting that ERK1/2 phosphorylation may underpin the observed functional adaptations.

## 4 Discussion

The gut microbiota is an integral regulator of host physiology, critically shaping the development and maturation of both innate and adaptive immune systems through the production of bioactive molecules that modulate systemic inflammation and immune responses. Bidirectional communication between the gut microbiota and the CNS via the gut–brain axis has emerged as a key determinant of neuroimmune homeostasis, with broad effects on brain development, plasticity, and function through the lifespan (28, 29, 49). Microglia are uniquely positioned to integrate gut-derived cues, given their high sensitivity to environmental changes and their capacity for long-lasting functional adaptation (7, 31). Perturbations in gut microbial composition or metabolite availability have been shown to markedly influence microglial morphology, metabolism, and inflammatory capacity, underscoring their dynamic tuning by peripheral signals rather than acting as static responders (31, 49, 50). Such calibration may imprint durable memory-like immune states, which become particularly relevant during repeated or chronic inflammatory exposure, where maladaptive microglial activation can accelerate neuronal dysfunction and disease progression.

While soluble microbial metabolites such as SCFA have received considerable attention as mediators of microbiota–brain communication, other classes of microbial signals remain less explored. In this regard, small EVs (diameter 30–150 nm) trivially also recognized as exosomes, released by the gut microbiota represent an emerging and potentially powerful mode of host–microbe interaction (32, 49, 51). These nanoscale lipid bilayer vesicles carry complex bioactive cargo and exhibit the capacity to cross epithelial and vascular barriers, positioning them as candidates for direct modulation of CNS immune cells (32, 34). In the present study, we provide evidence that gut-derived small EVs can induce long-term reprogramming of primary murine microglia toward a state of trained innate immune tolerance *in vitro*.

The gut-microbiota derived EVs represent a heterogeneous mixture, potentially comprising vesicles derived from both host cells and gut microbes (52, 53). Given that the gut microbiota is largely composed of trillions of commensal bacteria, these preparations are likely enriched in bacterial vesicles, including outer membrane vesicles (OMVs) from Gram-negative species and vesicles from Gram-positive bacteria, alongside host-derived vesicles (53, 54). Host-derived small EVs are characterized by the expression of canonical tetraspanin proteins such as CD9, CD63, and CD81, which play key roles in cargo sorting, membrane organization, cell adhesion, signaling, and vesicle fusion (55, 56). While bacterial EVs lack on tetraspanins, they contain species-specific components such as endotoxins, LTAs, and microbial proteins that can modulate host immune responses (57, 58). In accordance with the MISEV 2018/2023 guidelines, vesicles in the present study were isolated using size-exclusion chromatography, a strategy that limits physical stress and preserves vesicle integrity (39, 40). Compared with ultracentrifugation-based approaches, this method has been shown to better maintain structural fidelity and functional activity of small EVs, which is particularly important when interrogating immunological outcomes (59, 60). The gravity-driven separation and short processing time further minimize exposure to shear forces and adverse conditions, supporting the biological relevance of downstream functional analyses (60, 61). Consistent with these properties, our small EVs isolated from the murine gut microbiota exhibited characteristics of a mixed population of host and bacterial origin, highlighting the complexity of microbial–host vesicle crosstalk in the gut environment.

Emerging evidence indicates that microglia are capable of acquiring long-lasting, memory-like functional states in response to environmental cues. Numerous studies have shown that various PAMPs, such as low-dose LPS or β-glucan (*Candida albicans*), as well as viral mimetics (e.g., poly (I:C)), can reprogram microglia toward a trained pro-inflammatory phenotype, enhancing antimicrobial activity and potentially exacerbating neuroinflammatory disorders, including Alzheimer’s disease (24, 25, 36, 62). Moreover, endogenous danger signals like high-mobility group box 1 (HMGB1) or amyloid-beta (Aβ), may similarly promote aberrant microglial activation, leading to excessive synaptic pruning and neuronal dysfunction (26, 63, 64). Contrary, higher doses of LPS or β-glucan, as well as Aβ oligomers, can induce trained innate immune tolerance, characterized by attenuated pro-inflammatory responses and reduced oxidative stress, which may mitigate the severity of neurodegenerative disorders with inflammatory components (24, 44, 65, 66). Consistent with this, our data extend this concept by showing that small EV-primed murine microglia adopt an anti-inflammatory long-term state, with decreased production of TNF-α, IL-6, IL-1β, MCP-1, and ROS, alongside increased IL-10 levels. Interestingly, the effects of gut-derived small EVs appeared to be concentration-dependent. Previous studies have shown that low-intensity stimulation by microbial ligands promotes trained immunity through phosphoinositide 3-kinase (PI3K)/mechanistic target of rapamycin (mTOR)-driven metabolic reprogramming, increased glycolysis and glutaminolysis, and activating histone modifications such as H3K4me1 and H3K4me3 (15, 67, 68). In contrast, stronger or sustained stimulation can induce immune tolerance, a state associated with AMP-activated protein kinase (AMPK) activation, enhanced fatty acid oxidation and oxidative phosphorylation, and epigenetic remodeling that limits subsequent inflammatory responses (15, 67, 68). These mechanisms may underlie the concentration-dependent effects of small EVs observed in our study. While LPS-induced memory responses are largely mediated peripherally through TLR4 on meningeal and endothelial cells due to limited blood–brain barrier penetration, small EVs may access the CNS directly via transcytosis or paracellular routes, or indirectly through modulation of endothelial and perivascular immune cells, thereby influencing microglial signaling and functional programming (69–72). Gut microbiota metabolites, especially SCFA such as acetate and butyrate, have been reported to modulate microglial maturation, morphology, and function, reducing pro-inflammatory cytokine production (TNF-α, IL-1β, IL-6) in a model of sepsis-associated encephalopathy, thereby eliciting neuroprotective effects (31, 73). To date, EVs derived from commensal gut bacteria (i.e., *Lacticaseibacillus*, *Akkermansia*, *Clostridium*) have been shown to shift polarization of cells toward a tolerant phenotype, providing protection in mouse models of ulcerative colitis (74–76). Consistent with these findings, bacterial EVs derived from the gut microbiota have been shown to drive microglia toward a less inflammatory phenotype while enhancing phagocytic efficiency (34). To clarify the vesicle source underlying the observed immunomodulatory effects, we compared small extracellular vesicles derived from murine colorectal epithelial cells with bacterial vesicles from *E. coli* and *L. rhamnosus*. While epithelial small EVs did not affect basal cytokine secretion and enhanced responses upon secondary stimulation, bacterial vesicles suppressed TNF-α and IL-6 production following restimulation. These findings suggest that the observed immune-tolerant phenotype is primarily mediated by bacterial vesicles and support their role in long-term microglial reprogramming. Given that bacterial vesicles carry diverse proteins, lipids, metabolites, and nucleic acids with immunomodulatory potential (77, 78), future studies combining microbial source tracking with comprehensive cargo profiling will be required to define the mechanisms governing microglial reprogramming and immune tolerance. Moreover, Huang et al. reported that the gut microbiome profoundly modulates microglial transcriptomes and subpopulation dynamics, implicating microbial cues in the pathogenesis of neurodegenerative disorders (79). Overall, these findings support the concept that gut-derived signals, including SCFAs and small EVs, can induce functional reprogramming of microglia, shaping CNS immune homeostasis and inflammatory responsiveness.

Typically, memory-like inflammatory programs in innate immune cells are initiated by the sensing of pathogen-associated or endogenous danger signals through pattern-recognition receptors, thereby modulating NF-κB activity and inflammatory mediator production upon subsequent challenges (80, 81). Given the predominantly bacterial composition of gut–derived small EVs, we examined TLR-dependent signaling, as vesicle-associated LPS and LTA from Gram-negative and Gram-positive bacteria engage TLR4 and TLR2, respectively, while host-derived vesicles likely modulate innate pathways indirectly via associated proteins or nucleic acids (15, 45). Our data indicate that small EV–induced tolerance in murine microglia is linked to dampened MyD88-dependent TLR2 and TLR4 signaling, accompanied by reduced NF-κB activity downstream of p38 MAPK signaling, suggesting selective modulation of proximal inflammatory pathways rather than an impairment of microglial viability. Likewise, previous reports demonstrated that attenuated TLR4- and TLR2-MyD88-dependent signaling is linked with declined expression of pro-inflammatory mediators (i.e., TNF-α, IL-6, IL-1β) (45, 82, 83). Furthermore, negative regulators of innate immune signaling, including SHIP, IRAK-M, SOCS1, and A20, have been shown to contribute to the establishment of immune tolerance by suppressing TLR-mediated pathways and thereby limiting pro-inflammatory responses (15, 84–86). Stringent control of NF-κB p65 transcriptional activity by p38 MAPK signaling is critical to ensure a balanced innate immune response (87, 88). Concurrently, increased ERK1/2 activation observed in tolerant microglia provides a plausible mechanism for sustained IL-10 production, as ERK1/2 signaling is known to promote activator protein (AP)-1–dependent transcriptional programs that favor anti-inflammatory and regulatory gene expression, particularly under conditions of attenuated NF-κB activity (89, 90). Tight regulation of neuroinflammatory responses is essential to protect brain tissue from damage caused by excessive inflammation, and the trained innate immune tolerance induced by small EVs likely reflects such a regulatory program.

Long-term memory–like adaptations of the innate immune system are tightly associated to metabolic reprogramming and epigenetic regulation (14, 15, 91). In particular, trained innate immune tolerance has been associated with a shift away from pro-inflammatory bioenergetic states, characterized by restrained glucose metabolism (92–94). Impaired glycolysis has emerged as a key metabolic feature underlying attenuated inflammatory responses in innate immune cells (95–97). In line with this concept, our data demonstrate that small EV–primed murine microglia exhibit suppressed glycolytic activity, reflected by reduced expression of key glycolytic enzymes, including HK-2, PFK1, and PFKFB3, which collectively regulate glucose flux and lactate production. This metabolic restraint was accompanied by decreased lactate levels and correlated with dampened pro-inflammatory cytokine production. Consistent with our findings, previous studies have reported that LPS-tolerant innate immune cells, including microglia, display reduced glycolysis and altered cellular respiration, resulting in diminished adenosine triphosphate (ATP) generation and sustained immune tolerance (38, 98, 99). Such metabolic adaptation may directly shape the epigenetic landscape, as diminished glycolytic activity and lactate production constrain histone lactylation marks (i.e., H3K18la) and influence activating chromatin modifications (i.e., H3K4me3, H3K27ac), thereby reinforcing long-term suppression of pro-inflammatory gene expression (100–102). Here, we demonstrate that small EV priming induced a repressive epigenetic profile in murine microglia, characterized by reduced expression of activating histone H3 marks (H3K4me3 and H3K27ac) and increased H3K9me3, consistent with sustained suppression of pro-inflammatory gene programs. Concomitant reduction of H3K18 lactylation by small EV-priming further links diminished glycolytic flux and lactate availability to epigenetic reinforcement of trained innate immune tolerance in murine microglia, thereby constraining transcriptional programs associated with inflammatory activation and reinforcing a tolerant phenotype. Although direct causality was not tested, these findings are consistent with a model in which metabolic reprogramming and diminished lactate-driven histone lactylation jointly contribute to long-term immune tolerance in microglia (101, 103, 104). Activating histone modifications such as H3K4me3 and H3K27ac are well established markers of accessible chromatin that facilitate transcription factor binding, including NF-κB, supporting inflammatory gene expression (105, 106). Emerging evidence indicates that the gut microbiota and its metabolites, particularly SCFAs such as acetate, act as important modulators of these epigenetic states, contributing to long-term memory-like features of microglia that might be implicated in recurrent depressive-like events in mice (107, 108). During inflammaging, sustained overactivation of inflammatory gene programs has been linked to broad enrichment of H3K27ac across gene bodies, whereas reduction of this acetylation attenuates neuroinflammatory signaling (109, 110). Notably, Huang et al. demonstrated that suppression of H3K27ac disrupts innate immune memory formation and dampens inflammatory responses upon secondary stimulation, highlighting its role in maintaining chronic neuroinflammation (111). Histone lactylation at H3K18, driven by HK-2–dependent glycolysis and lactate accumulation, has likewise been implicated in supporting memory-like inflammatory programs, although this modification may also promote anti-inflammatory events in specific contexts such as focal cerebral ischemia (112–114). In contrast, elevated H3K9me3, often mediated by the activity of methyltransferases, represents a repressive chromatin state that limits inflammatory gene transcription and has additionally been linked to neuroprotective and repair-associated processes involving brain-derived neurotrophic factor signaling (115–118). These findings position gut–derived small EVs as modulators of microglial immune memory by coupling metabolic restraint with coordinated epigenetic repression, thereby stabilizing a tolerant, neuroprotective microglial phenotype.

Alongside their inflammatory, and metabolic reprogramming, microglia rely on functional behaviors such as migration and phagocytosis, which are essential for responding to tissue damage and maintaining CNS homeostasis during neuroinflammatory events. Despite a restrained inflammatory profile, small EV–primed murine microglia displayed increased migratory and phagocytic activity *in vitro*, accompanied by elevated expression of the phagocytic receptor CD32 and the adhesion molecule CD11a, a phenotype that may be regulated independently of pro-inflammatory transcription by ERK1/2 signaling. Similarly, an increasing body of evidence demonstrates that LPS preconditioning, mediated through RelB-dependent epigenetic silencing of H3K4me3, enhances microglial phagocytic activity and thereby attenuates cerebral β-amyloid accumulation in a mouse model of Alzheimer’s pathology (24, 119). In addition, peripheral immune stimulation has been shown to modify pathological outcomes after stroke, underscoring the contribution of microglial innate immune memory to key disease features in experimental models, in line with observations reported in clinical settings (10, 120, 121). An anti-inflammatory microglial phenotype characterized by enhanced ERK1/2 signaling and elevated expression of the Fcγ receptor CD32 has been reported to preserve or even promote phagocytic capacity, thereby supporting neuroprotective functions in the CNS, while concomitant upregulation of the adhesion molecule CD11a and ERK1/2-dependent cytoskeletal regulation contributes to increased microglial motility, as observed in experimental models associated with neurodegenerative disorders (122–126).

Several limitations should be considered when interpreting these results. First, our experiments in primary microglia under controlled *in vitro* conditions cannot fully capture the complexity of the *in vivo* brain, including interactions with neurons, astrocytes, endothelial cells, and peripheral immune inputs. Moreover, this reductionist culture system does not recapitulate the dynamic gut–brain axis, including systemic circulation, blood–brain barrier regulation, and immune cell trafficking. Therefore, the observed effects of gut-derived small EVs on microglial immune memory cannot be directly extrapolated to physiological or pathological conditions *in vivo*. Future studies employing animal models will be necessary to validate whether small EV–mediated microglial tolerance occurs in the intact organism and to determine its functional relevance in health and disease. Secondly, although we demonstrate robust functional and molecular effects of small EV priming, the specific vesicular gut microbes- or host-derived (i.e., proteins, lipids, nucleic acids) components responsible for tolerance induction remain unresolved and it represent a highly challenging adventure to be solved. Thirdly, the long-term stability of small EV–induced tolerance and its relevance to neurodegeneration, chronic neuroinflammation, aging, and early-life immune challenges remain unclear and warrant further investigation in longitudinal *in vivo* studies. Finally, indirect effects of the gut microbiota via hormonal, vagal, or cytokine signaling pathways can directly affect microglia function. As gut–brain communication involves multiple interconnected immune, neural, and endocrine pathways, the direct effects observed in our microglial cultures likely represent only part of the overall biological response. Future studies should therefore examine how gut-derived small EVs interact with these signaling networks to modulate microglial function *in vivo.* However, the use of such a heterogeneous mixture of gut–derived small EVs may provide a more physiologically relevant approach to investigate their role in modulating microglial function and CNS immune homeostasis.

Collectively, our findings expand the concept of the gut–brain axis by identifying gut-derived small EVs as active regulators of microglial immune memory. While previous studies have emphasized soluble microbial metabolites, our data identify gut–derived small EVs as a sustained microbiota–host signaling route in the CNS that induces trained innate immune tolerance, characterized by restrained TLR–p38 MAPK–dependent inflammation, reduced glycolysis and activating histone marks, yet preserved ERK1/2-dependent microglial migration and phagocytosis.

## 5 Conclusions

These findings identify a previously underexplored mechanism by which the gut microbiota shapes memory-like immune responses in murine microglia within the CNS. We show that gut–derived small EVs reprogram primary murine microglia toward a tolerant, memory-like state marked by restrained inflammatory signaling coordinated by metabolic and epigenetic alterations, alongside preserved essential microglial functions *in vitro*. However, these observations are based on an *in vitro* microglial model and therefore cannot be directly extrapolated to *in vivo* physiological or pathological conditions. Given that chronic microglial activation drives neurological disease while broad immunosuppression entails substantial risk, elucidating vesicle-mediated microbiota–brain communication across physiological and pathological contexts may open new therapeutic avenues for neuroinflammatory disorders. Further *in vivo* and preclinical studies will be required to determine whether these mechanisms are maintained in the intact brain and whether they hold therapeutic relevance for neuroinflammatory disorders.

## Data Availability

The datasets used in the current study are available from the corresponding author on reasonable request.
