# Supplementary material for: Gut-derived small extracellular vesicles support trained innate immune tolerance in murine microglial cells
Source: Front Immunol. 2026 Aug 6;17:1858954. doi: 10.3389/fimmu.2026.1858954 (PMC13489878; doi:10.3389/fimmu.2026.1858954)
Supplement: Supplementary file 1 [file DataSheet1.docx]

Supplementary Material

# 1 Supplementary Figures

**Supplementary Figure 1.** **Effects of bacterial- and colorectal-derived small EVs on inflammatory responses in murine primary microglia.** Primary microglia were exposed for 24 h to small EVs derived from colorectal epithelial cells (CMT-93; 50 µg/mL), Gram-negative bacteria (*E. coli*; 10 µg/mL), or Gram-positive bacteria (*L. rhamnosus*; 25 µg/mL). Following stimulation, cells were washed and allowed to recover in fresh medium with regular medium replacement. Subsequently, microglia were challenged with LPS (100 ng/mL), and samples were collected at the indicated time points for further analyses. Concentrations of TNF-α and IL-6 in culture supernatants were determined by ELISA 24 h after either the initial small EV stimulation (A, B; n = 4) or the secondary LPS challenge (C, D; n = 4) and normalized to total protein content. Data were presented as scatter dot plots, mean ± SEM, 1-Way ANOVA (Holm-Sidak post-hoc), *p<0.05, * indicating statistical significance compared to (B, C) unstimulated or (D, E) unprimed control.

**Supplementary Figure 2.** **Validation of primary microglial culture purity and effects of small EVs on cell viability and cytotoxicity.** Primary microglial cultures were established and evaluated to verify cellular purity prior to *in vitro* experimentation. Microglial enrichment was determined by immunocytochemical labeling of the microglial marker IBA1, with nuclei counterstained using DAPI (total cells, blue color). The proportion of (A, n = 5) IBA1-positive cells relative to total cell number was quantified from at least five randomly selected microscopic fields per preparation. To assess potential effects of small extracellular vesicles (small EVs; 1 or 3.5 µg/mL) on cell health, microglial cultures were treated according to the two-step stimulation protocol and the (B, n = 5) cell viability was assessed using an MTT-based viability assay, while (C, n = 6) cytotoxicity was evaluated using a Abcam colorimetric assay. Data were presented as scatter dot plots, mean ± SEM. No significant changes in viability or cytotoxicity were observed compared with unstimulated control cells.
